# China's “dynamic clearing” epidemic prevention policy: Achievements, challenges, and prospects

**DOI:** 10.3389/fpubh.2022.978944

**Published:** 2022-09-14

**Authors:** Quansheng Wang, Lansong Huang

**Affiliations:** Law School, Shandong University, Weihai, China

**Keywords:** public health emergency, dynamic clearing, external input prevention and internal rebound prevention, people's lives and health, digital governance

## Abstract

The COVID-19 outbreak makes up a major public health emergency, and each country has adopted different epidemic prevention policies and measures. Since the control of COVID-19 in 2020, China has gradually developed a “Dynamic Clearing” epidemic prevention policy of “external input prevention and internal rebound prevention”. The policy has been effective in protecting people's lives and health and developing the country's economy as much as possible, but it has also faced some challenges, such as slowing economic development, huge prevention, and control costs, and expanding secondary disasters and risks. Reviewing China's current “dynamic clearing” policy, it is still the overall policy to continuously improve guiding policies, construct scientific prevention measures, and promote digital governance at the grassroots level.

## Digital governance

It has been over 2 years since the outbreak of COVID-19 occurred, and the epidemic still shows no signs of abating. In particular, since March 2022, concentrated outbreaks have occurred in several Chinese cities such as Qingdao, Weihai, Jilin, Changchun, Shenyang, Shanghai, Guangzhou, Chengdu, Beijing, and so on, with 534, 8,157 confirmed cases and 24,034 cumulative deaths reported nationwide as of 16:00 on 8 August (1, 2). In Shanghai, in particular, since the beginning of March, the epidemic has become more and more serious. On 1 April, a region-wide standstill began, with traffic and supply chains blocked until 16 May, when it was gradually lifted, and on 1 June, normal production and living order was fully restored, and regular management of the epidemic prevention and control was implemented. As the largest city in China and the third-largest in the world, with a population of more than 27 million, it plays a pivotal role in China's economic development. In 2021, Shanghai's total economic output ranked 10th among the country's 31 provinces and cities, and the sealing control in the past 2 months has had a profound impact on the economy of Shanghai and China.

Faced with this major public health emergency, China has been adopting the “external prevention of importation and internal prevention of rebound” approach after the battle against the epidemic in Wuhan at the beginning of 2020, and although there have been sporadic occurrences, they have been promptly and effectively controlled and have had little impact. On December 11, 2021, the National Health Commission formally proposed “dynamic clearing” as the general policy for the prevention and control of epidemics in China. On 5 March 2022, in the Fifth Session of the 13th National People's Congress, He Lifeng, chairperson of the National Development and Reform Commission, presented the Report on implementing the National Economic and Social Development Plan for 2021 and the Draft National Economic and Social Development Plan for 2022, also proposed the general policy and guideline of “dynamic clearing” for epidemic prevention, which was hotly debated by the delegates of the National People's Congress. According to an article entitled “Why the world needs China's dynamic clearing policy” published on the Bloomberg website on 9 February this year, China's “dynamic clearing policy” has benefited the world. The Wall Street Journal website also published an article on 16 February entitled “China's new “dynamic clearing” policy on infections contains lessons for other countries,” stating that China's “dynamic clearing” anti-epidemic policy has achieved what every country was seeking for 2 years: low death rate and as little economic disruption as possible (3). On March 17, General Secretary Xi Jinping chaired a meeting of the Standing Committee of the Central Political Bureau, stressing that “we should always adhere to the supremacy of the people and life, adhere to scientific precision and dynamic clearance, and curb the momentum of the spread of the epidemic as soon as possible,” further showing the direction for the prevention and control of the epidemic (4). During his visit to Hainan on April 10–13, Xi Jinping once again stressed the importance of “adhering to scientific precision and dynamic clearing” (5). Therefore, the policy of “prevention of external importation and internal rebound” under the general policy of “dynamic clearing” is still the basic platform and guideline for future epidemic prevention work.

Liang Wannian, head of the expert group of the National Health and Wellness Commission's Leading Group for Epidemic Response and Disposal, introduced the so-called dynamic clearing, which includes three aspects: the first is the timely and proactive detection of sources of infection. The sources of infection include patients, asymptomatic infected people, and other animals that may carry pathogens. The main means of detecting the sources of infection are through monitoring early warnings at fever clinics and some tests and active screening. Second, when a case is detected, public health and social interventions are quickly taken, including control of the outbreak site, the management of close contacts, epidemiological investigation, and control measures to reduce crowd gathering. Third, effective treatment, mainly using a combination of Chinese and Western medicine and other methods, to effectively treat patients, stop the progress of the epidemic as soon as possible and far, prevent light from becoming serious, and reduce the occurrence of serious illness and death. Liang Wannian stressed that the general policy of “dynamic Clearing” is to pursue the greatest possible integration of socioeconomic development and epidemic prevention and control. The “dynamic clearing” approach requires speed and precision, and the rapid elimination of outbreaks when cases are detected (6).

Over the past 2 years since the outbreak of COVID-19, China has provided the world with a uniquely Chinese experience and solution to combat the epidemic, which is the “dynamic clearing” policy of “prevention of external importation and internal rebound.” The outstanding achievements of this epidemic prevention policy are 2–fold.

First, it has protected the lives and health of the people. The Communist Party of China has always put “people first, life first” as its governing philosophy. China has withstood round after round of epidemics and dealt with dozens of localized epidemics quickly and effectively, protecting people's lives and health to the maximum extent possible. As of 6:30 p.m. Beijing times on May 25, 2022, there were 52,867,060 confirmed cases of NCCP and 630,059 deaths worldwide. In the United States, there were 85,145,035 cumulative confirmed cases of NCCP and 1,029,269 cumulative deaths (7), see Table 1.

**Table 1:** Confirmed cases and deaths of new coronary pneumonia worldwide, USA and China.

	**Cumulative number of confirmed cases of new coronary pneumonia**	**Cumulative new coronary pneumonia deaths**
Worldwide	528670960	6303059
United States	85145035	1029269
China	2114305	161515

According to the WHO's ranking of 194 countries or territories worldwide in terms of data on number of excess new crown deaths, the top five countries are India, Russia, Indonesia, the United States, and Brazil, with a death toll of 4740894; 1072326, 1028565, 932458, and 681 1,267, respectively. The country with the lowest number of deaths was China, ranked 194th, followed by Japan (8), see Table 2.

**Table 2:** Global ranking of excess deaths from new coronary pneumonia in 194 countries.

**Rank**	**Name of the country**	**Excess deaths in New coronary pneumonia**
1	India	4740894
2	Russia	1072326
3	Indonesia	1028565
4	United States	932458
5	Brazil	681267
……	……	……
193	Japan	−19471
194	China	−52063

However, according to a paper published in The Lancet in March 2022, *Estimating excess mortality because of the COVID-19 pandemic: a systematic analysis of COVID-19-related mortality, 2020-21* (9) reveals that while the official number of deaths recorded for the period 1 January 2020 to 31 December 2021 is 5.9 million, the new study estimates that 18.2 million additional deaths (excess deaths) occurred during the same period. On the country level, the highest estimated excess deaths are in India (4.1 million), the United States (1.1 million), Russia (1.1 million), Mexico (798,000), Brazil (792,000), and Indonesia (736,000), and Pakistan (664,000), see Table 3.

**Table 3:** Ranking of excess deaths from new coronary pneumonia by country.

**Rank**	**Name of the country**	**Deaths (10,000)**
1	India	410
2	United States	110
3	Russia	110
4	Mexico	79.8
5	Brazil	79.2
6	Indonesia	73.6
7	Pakistan	66.4

A new concept invoked by the WHO here is that of “excess mortality”, which refers to the difference between the number of recorded deaths from all causes and the number of deaths expected based on past trends, it is a key indicator of the true number of deaths in a pandemic. Various studies have shown that geographical location, population density, demographics, prosperity, etc. can all influence excess mortality (10–14).

We can argue that regardless of the data published by either party, the number of deaths in China in the COVID-19 is tiny and the rate is low. From this perspective, China is focusing on protecting people's lives and health in its epidemic prevention.

The dynamic clearance effectively safeguards sustained and stable economic development. The key to determining the best solution to combat the epidemic lies in how to contain it in a shorter period at a lower cost, reduce the adverse impact brought about by the epidemic, and ensure sustained healthy and stable economic and social development with good prevention and control results. In 2020, China will be “the first to control the epidemic,” “the first to resume production” and “the first to turn economic growth from negative to positive.” In 2021, GDP will grow by 8.1% compared to the previous year, with an average growth rate of 5.1% over the next two years, making it one of the fastest-growing economies in the world. China has stabilized its supply chain in 2020 and 2021, maximizing the smooth flow of the supply chain; stabilizing the industrial chain, enabling the rapid recovery and stable development of the Chinese economy; and stabilizing consumer confidence, creating favorable conditions for the market to maintain long-term stability (15).

However, it should also be noted that China's “dynamic clearing” disease prevention policy also faces serious challenges.

First, the economic growth situation is not optimistic. The central government has established an expected growth target of around 5.5% for the entire year of 2022 in China. The epidemic affected the economy, by 4.8% year-on-year in the first quarter of this year, a large distance from the expected target. In April, the value-added of industries above the national scale fell by 2.9% year on year. The national services production index fell by 6.1% year-on-year in April. Total retail sales of consumer goods fell by 11.1% year on year to RMB2, 948.3 billion in April. Fixed asset investment (excluding farm households) fell by 0.82% in April from a year earlier. Influenced by the intensifying downward pressure on the economy, the urban survey unemployment rate continued to climb in April, reaching 6.1%, widening the gap with the expected target of 5.5% or less for the year. Among them, the survey unemployment rate for the population aged 16–24 climbed to 18.2% (16).

Second, the cost of epidemic prevention continues to increase. New coronavirus mutations are more deeply hidden and spread quickly, making prevention difficult. Some economists have calculated the cost of prevention and control in Shanghai from April 1st to May 22nd.

The direct cost of the 52 days was RMB 99.2 billion, or RMB 1.907 billion per days on average, from April 1, when the official announcement was made that the entire area would be under static management, to May 22, when some bus lines resumed operation. The implicit (indirect) cost test result was $3.97 trillion, or an average of $76.3 billion per days. The direct and implicit costs add up to RMB 4.07 trillion, which is close to the annual GDP of Shanghai in 2021 (RMB 4.32 trillion) (17). See Tables 4, 5.

**Table 4:** Summary of the direct costs of shanghai seal control.

	**Government/Aid (parity)**	**Residents (rush price)**	**Total (RMB billion)**
Subsistence expenditure	100	300	400
Expenditure on nucleic acid/antigen tests	78		78
Square cabin hospital construction/renovation expenditures	150		150
Community volunteer allowance expenditure	331		331
Roadblocks and other expenses	33		33

**Table 5:** Summary of hidden (Indirect) costs of shanghai sealing control (RMB billion).

**Rent, utilities, and gas costs**	**1,021**
Nucleic acid waiting for costs	1,348
Loss of industrial output	1,473
Loss of market capitalization of securities markets	35,700
Secondary hazards and others	147
Total	39,689

Apart from Shanghai, in other cities in China such as Beijing, Guangzhou, Changchun, Shenyang, and other cities where epidemics have occurred, the costs of prevention and control are enormous. The costs of prevention and control will continue to increase, putting enormous financial pressure on local governments at all levels.

Third, secondary disasters and risks have expanded. In some places, in order to cut off the source of transmission and prevent the spread of the epidemic, simple and violent methods such as road blocking and road digging were adopted to control the movement of people, which affected people's normal travel and life and was not conducive to scientific epidemic prevention and control; the public refuses to seek medical attention for fear of cross-infection and resorts to self-isolation at home instead of going to the hospital, allowing family members to infect each other and leading to an increase in family clusters of cases; some local hospitals have adopted a one-size-fits-all approach, treating only patients with COVID-19 and neglecting the needs of other patients, resulting in many non-COVID-19 patients not receiving timely treatment; some residents go out without protective gear, leading to an increased chance of transmission; Sometimes, the improper use of protective equipment for epidemic control has led to a high number of accidents. We should give these secondary disasters arising from epidemic control sufficient attention.

Although there are indeed serious challenges to the “dynamic clearing” epidemic prevention policy, there is still a need to take stock of past epidemic prevention experiences, reflect on the current epidemic prevention policy and its implementation measures, and promote the scientific of China's epidemic prevention measures.

First, the “dynamic clearing” epidemic prevention policy should be further improved. The National Health Commission, as China's public health authority, should convene experts from various fields, including clinical medicine, law, public health management, immunology, etc., to conduct scientific discussions and ensure the scientific and rational nature of the policy. The basic guiding principle is that on the premise of adhering to the general policy of “dynamic clearing,” some localities can explore and summarize some good experiences and practices according to the local epidemic situation and characteristics, to further improve the scientific and precise level of epidemic prevention and control, and strive to achieve the maximum prevention and control effect with the minimum cost.

Second, vaccination should be strengthened, especially for the elderly and children. According to the mortality rates in various countries, the elderly and children are the biggest victims of the epidemic, as their immunity is low, their organs are declining and aging, and children's organs are underdeveloped. The vaccination rates in China is currently 88.64%, but the rate for the elderly is only 81.49% (2). The need to further increase the vaccination rate for the whole population as well as the full vaccination rate is a fundamental measure to prevent the attack of the new coronavirus.

Thirdly, we should promote the science of epidemic prevention and control measures. China has a vast territory, and we must not apply prevention and control measures across the board. We must firmly prevent and avoid the tendency to “relax prevention and control” and “over-prevention and control.” Because of the new characteristics of Omicron, we should further strengthen containment, management, and control, shorten the time of centralized isolation, and implement categorized treatment. Only by targeting and scientifically and precisely preventing the epidemic can we achieve the greatest effectiveness in prevention and control at the least cost.

Fourth, it should promote the construction of digital government, especially to strengthen the digital governance of the grassroots' government. One of the major reasons for the many problems in the prevention and control of the Newcastle pneumonia epidemic in some areas in terms of epidemic risk warning, information collection, material deployment, and staff coordination is the lagging construction of digital government, insufficient application of digital technology, and the need to improve the efficiency of digital governance. Government departments at all levels should use digital technologies such as big data, artificial intelligence, and cloud computing to play a better supporting role in the epidemic (risk) monitoring and analysis, virus (crisis) tracing, prevention, control, and treatment, and resource deployment. In the fight's context against the epidemic, the government should speed up the reform of grassroots services, vigorously optimize the “Internet + government services,” focus on matters that are frequently used by the public, and promote “one network for all” government services, so that the public can conduct business without having to leave home. The government will also focus on the maintenance and promotion of government service apps, so that they are not only usable but also useful, and strive to make “contactless services” a standardized work requirement so that the “palm of the hand” becomes a personal service for the masses. The mainstream service mode of government services.

## Author contributions

Conceptualization and writing—review and editing: QW. Methodology: QW and LH. Formal analysis: LH. Writing—original draft preparation: LH. All authors have read and agreed to the published version of the manuscript. All authors contributed to the article and approved the submitted version.

## Conflict of interest

The authors declare that the research was conducted in the absence of any commercial or financial relationships that could be construed as a potential conflict of interest.

## Publisher's note

All claims expressed in this article are solely those of the authors and do not necessarily represent those of their affiliated organizations, or those of the publisher, the editors and the reviewers. Any product that may be evaluated in this article, or claim that may be made by its manufacturer, is not guaranteed or endorsed by the publisher.

## References

[1] Baidu. Epidemic Real-Time Big Data Report. Available online at: https://voice.baidu.com/act/newpneumonia/newpneumonia/?from=osari_aladin_banner (2022). (accessed August 8, 2022).

[2] Li Z. National Health Commission: the whole new crown vaccination accounted for 88.64% of the total population of the country, the elderly vaccination rate gradually increased. Available online at: https://www.yicai.com/news/101397655.html (2022). (accessed May 26, 2022).

[3] Chen X Hu Z Liang JQ Song C Li H. The experience and significance of China's fight against the epidemic over the past two years. Xinhua Daily Telegraph. (2022) 14:5. 10.28870/n.cnki.nxhmr.2022.003168

[4] Xinhua News Agency. Xinhua Review: China's “dynamic Clearing” Focus Observation. Available online at: https://baijiahao.baidu.com/s?id=1729723279842295826&wfr=spider&for=pc (2022). (accessed May 26, 2022).

[5] People's Daily Commentator. Adhering to Scientific Accuracy Dynamic Clearance. Beijing: People's Daily Publishing House (2022). Available online at: https://www.sohu.com/a/538340886_119038 (accessed August 23, 2022).

[6] Liang WN. China's “Dynamic Clearing” has Three Sub-targets. Available online at: https://baijiahao.baidu.com/s?id=1731414458753611972&wfr=spider&for=pc (2022). (accessed May 26, 2022).

[7] Zhan G. S. How much does it cost to seal control in Shanghai? (2022). Available online at: https://3g.163.com/dy/article/H878UUB905522S1P.html (accessed May 26, 2022).

[8] WHO. WHO Publishes Ranking of the Number of New Crown Deaths Worldwide in 2020 and 2021. Available online at: https://www.163.com/dy/article/H79TJI1O054488DF.html (accessed May 26, 2022).

[9] Wang H Paulson KR Pease SA Watson S Comfort H Zheng P. Estimating excess mortality due to the COVID-19 pandemic: a systematic analysis of COVID-19-related mortality, 2020–21. Lancet. (2022) 399:1513–36. 10.1016/S0140-6736(21)02796-3 35279232 PMC8912932

[10] Achilleos S Quattrocchi A Gabel J Heraclides A Kolokotroni O Constantinou C. Excess all-cause mortality and COVID-19-relatedmortality: a temporal analysis in 22 countries, from January until August 2020. Int J Epidemiol. (2022) 51:35–53. 10.1093/ije/dyab123 34282450 PMC8344815

[11] Ali AB Shaikh A Maghami N Zia M., Wolf DA, Bonville DJ. Impact of Covid-19 pandemic on volume and surgeon professional fees generated by emergency general surgery procedures. Surg Endos. (2022) 1–7. 10.1007/s00464-022-09168-z PMC8926383 35296948

[12] French G Hulse M Nguyen D Sobotka K Webster K Corman J. Impact of hospital strain on excess deaths during the COVID-19 pandemic — United States, july 2020–july 2021. MMWR. Morb Mortal Wkly Rep. (2021) 70:1613–6. 10.15585/mmwr.mm7046a5 34793414 PMC8601411

[13] Giannouchos TV Biskupiak J Moss MJ Brixner D Andreyeva E Ukert B. Trends in outpatient emergency department visits during the COVID- 19 pandemic at a large, urban, academic hospital system. Am J Emerg Med. (2021) 40:20–6. 10.1016/j.ajem.2020.12.009 33338676 PMC7725055

[14] Karlinsky A Kobak D. Tracking excess mortality across countries during the COVID-19 pandemic with the World Mortality Dataset. eLife. (2021) 10: e69336. 10.7554/eLife.69336 PMC8331176 34190045

[15] Xinhua News Agency. Xinhua Commentary: Adhere to the “dynamic Clearing”, do not Relax. Available online at: https://m.gmw.cn/2022-03/31/content_35625760.html (2022). (accessed May 26, 2022).

[16] Zhou MM, Zhou, XL,. Charting China's Economic Data in April. Available online at: https://www.sohu.com/a/547634956_121257854 (2022). (accessed May 26, 2022).

[17] Zhang GS,. How Much Does it Cost to Seal Control in Shanghai? Available online at: https://3g.163.com/dy/article/H878UUB905522S1P.html (2022). (accessed May 26, 2022).

